# Investigating the role of mechanics in lignocellulosic biomass degradation during hydrolysis: Part II

**DOI:** 10.1002/btpr.3083

**Published:** 2020-09-29

**Authors:** Ramūnas Digaitis, Emil Engelund Thybring, Lisbeth Garbrecht Thygesen

**Affiliations:** ^1^ Department of Geosciences and Natural Resource Management, Faculty of Science University of Copenhagen Frederiksberg C Denmark; ^2^ Biofilms Research Center for Biointerfaces Malmö University Malmö Sweden

**Keywords:** fiber attrition, lignocellulose depolymerization, mechanical agitation, reactor design and operation

## Abstract

Lignocellulose breakdown in biorefineries is facilitated by enzymes and physical forces. Enzymes degrade and solubilize accessible lignocellulosic polymers, primarily on fiber surfaces, and make fibers physically weaker. Meanwhile physical forces acting during mechanical agitation induce tearing and cause rupture and attrition of the fibers, leading to liquefaction, that is, a less viscous hydrolysate that can be further processed in industrial settings. This study aims at understanding how mechanical agitation during enzymatic saccharification can be used to promote fiber attrition. The effects of reaction conditions, such as substrate and enzyme concentration on fiber attrition rate and hydrolysis yield were investigated. To gain insight into the fiber attrition mechanism, enzymatic hydrolysis was compared to hydrolysis by use of hydrochloric acid. Results show that fiber attrition depends on several factors concerning reactor design and operation including drum diameter, rotational speed, mixing schedule, and concentrations of fibers and enzymes. Surprisingly, different fiber attrition patterns during enzymatic and acid hydrolysis were found for similar mixing schedules. Specifically, for tumbling mixing, slow continuous mixing appears to function better than faster, intermittent mixing even for the same total number of drum revolutions. The findings indicate that reactor design and operation as well as hydrolysis conditions are key to process optimization and that detailed insights are needed to obtain fast liquefaction without sacrificing saccharification yields.

## 1 INTRODUCTION

Lignocellulosic biomass is highly recalcitrant and its depolymerization into valuable precursors for bio‐based fuels and chemicals is a complex process. Standard depolymerization of biomass comprises several stages including biomass pretreatment, enzymatic hydrolysis as well as product purification or fermentation.^1^ Enzymatic hydrolysis is typically the central part of such a biorefining process, and in this step the majority of the carbohydrate polymers of the biomass is depolymerized into monomeric compounds, for example, glucose. It is of foremost importance to obtain a high product concentration during hydrolysis for the subsequent steps to be economically feasible. As a result, hydrolysis needs to be carried out at relatively high substrate‐to‐water concentration, usually above 15% insoluble solids.^2^, ^3^ Due to the hygroscopic nature of biomass, the amount of free water present in a hydrolysis reactor diminishes rapidly with increasing biomass concentration and, depending on the type of biomass, free water might be absent at concentrations above 15–40% insoluble solids.^4^, ^5^ Water is essential for biomass hydrolysis not only because it is involved in the hydrolysis reaction, but also as it acts as a dispersing agent for biomass particles and provides a diffusion medium for enzymes and soluble hydrolysis products.^6^ A limited amount or absence of free water during hydrolysis is therefore associated with limited hydrolysis efficiency and challenging processing.^4^, ^7^ This includes ineffective heat and mass transfer,^6^ end‐product inhibition,^8^ biomass agglomeration,^9^ decreased mixing efficacy and, at the same time, increased energy requirements for mixing.^10^, ^11^

Many different strategies have been used to increase biomass degradation rate, including the use of more efficient enzyme formulations,^12^ higher enzyme dosages,^13^ addition of supplemental agents (e.g., surfactants),^14^, ^15^ as well as improving reactor design and operation in order to maximize physical breakdown of biomass particles.^2^, ^16^ The importance of mechanical agitation during hydrolysis of lignocellulose is increasingly recognized.^9^, ^17^, ^18^ This is primarily due to the findings that enzymes and mechanical loading act synergistically in biomass degradation and that mixing can significantly affect biomass degradation rate.^2^, ^17^ In particular, it has been found that free fall agitation, that is, tumbling, where mixing relies on gravity, is much more effective than stirring for hydrolysis at high biomass concentration.^2^, ^16^

It is suggested that more efficient agitation promotes heat and mass transfer, including enzyme redistribution and dispersal of hydrolysis products away from the active sites of enzymes, hereby alleviating end product inhibition.^19^, ^20^ Notably, physical forces acting on fibers during mixing also directly contribute to fiber attrition.^9^, ^17^, ^20^ During tumbling mixing, the fibers are continuously subjected to the impact force which is generated once the falling fibers hit the bottom side of a reactor. Equation (1) defines the impact force of a falling object and indicates which parameters influence the impact force and thus physical fiber attrition, where *P* (N) specify impact force, *m* (kg) is object mass, *g* (m/s^2^) gravitational acceleration, *h* (m) fall height and *s* (m) slow down distance.

(1) $$P=\frac{m∙g∙h}{s}$$

Fiber attrition, that is, the reduction in size of fiber fragments during agitation, does not necessary lead to higher hydrolysis yields, although it increases the surface area and potentially enables higher surface coverage with enzymes.^9^, ^20^ However, shorter fibers are less prone to entangle.^21^ As a result, shortening of fibers during hydrolysis typically leads to liquefaction, that is, a less viscous fiber suspension, which can be pumped easier between different parts of the process equipment.^21^, ^22^ Consequently, the risk of pipe blockage is reduced. On the other hand, enzymes are sensitive biological molecules that can be negatively affected by process related factors, including mixing. Mixing induces shear forces and leads to aeration of enzymes, which accelerates enzyme inactivation.^23^, ^24^, ^25^ Due to the complex synergistic and antagonistic effects of agitation on biomass degradation, it is necessary to determine the optimal reactor design and operation conditions for achieving fast liquefaction and optimal hydrolysis yields.^9^

In the first part of this study we showed that neither mechanical agitation alone nor enzymatic treatment without mechanical agitation has any noteworthy effect on flax fiber attrition. It was found that successive treatment where enzymatic hydrolysis was preceded by mechanical agitation did not induce any substantial segmentation (i.e., attrition or shortening) of flax fibers. However, we showed that fibers subjected to prolonged hydrolysis where more susceptible to fracture during proceeding mechanical treatment than untreated fibers. Attrition of fibers was gradual, indicating a fatigue type of failure. Unlike individual or sequential treatments, simultaneous enzymatic and mechanical treatment was found to promote fast fiber shortening central for liquefaction. Higher hydrolysis yields, however, were obtained from non‐agitated samples after prolonged enzymatic treatment, indicating that mechanical agitation in the long run reduces activity of the cellulolytic enzymes, thus adding an antagonistic component to the interplay between mechanical agitation and enzymatic saccharification.^9^

The aim of the second part of the study, the findings of which are presented in this article, was to investigate how different mixing regimes during hydrolysis affect fiber attrition. Flax fibers were used as a model substrate, once again, and effects of reactor design and operation on fiber attrition during hydrolysis were evaluated based on three different laboratory‐scale agitation systems.

## 2 MATERIALS AND METHODS

Flax fiber bundles (*Linum usitatissimum* L.), obtained from the company Skytten (www.skytten-danmark.dk) and cut to ca. 4.5 mm segments were used as a model substrate. Flax was chosen because it is obtainable as a pure fibrous substrate composed of long, stiff fibers, whose length can to some degree be controlled by cutting into segments, and which have many dislocations, which make them prone to segmentation. Further, flax has a relatively high cellulose content compared to many other substrates, allowing for some saccharification to take place even for unpreprocessed material. To facilitate fast and accurate preparation of the fiber material, prior to cutting with a paper cutter, aligned fiber bundles were wetted in water and then physically stabilized by immersing into liquid nitrogen. In order to minimize the amount of short fibers and remove any inorganic debris, the fiber segments were thoroughly washed in demineralized water after cutting and driven through a set of sieves (aperture diameter of 3.15 and 1.4 mm) which mostly trapped long fibers. Finally, the flax fibers were dried at 60°C to app. 94% dry matter (DM) content. The chemical composition of the flax fibers was determined after washing to 60.7% glucan, 5.8% acid insoluble lignin, 0.4% xylan, 2.7% galactan, 0.6% arabinan, 2.7% manan, 12.0% EtOH extractives and 0.7% ash.^26^ Acid soluble lignin was not quantified in this study and this may be part of the reason why the mass balance of the composition analysis amounts to less than 100%.

Hydrolysis experiments were carried out using either the multicomponent Cellic CTec2 (Novozymes, Bagsværd, Denmark) enzyme preparation or hydrochloric acid. The initial (stock) enzyme concentration was determined with UV–Vis spectroscopy. Prior to UV–Vis measurements, enzymatic stock solution was diluted with Milli‐Q water to such an extent that absorbance was below 1. Enzymatic solutions of required concentration were then prepared by diluting stock solution with a 50 mM pH 4.8 sodium citrate buffer supplemented with 0.05% sodium azide, to prevent microbial growth. Unless stated otherwise, enzyme loading was 5.33 mg/g DM flax and the substrate (insoluble solids) loading was 25%. Acid hydrolysis was carried out using a 1.6 M HCl acid solution. Both acid and enzymatic hydrolysis experiments were carried out in 20 ml scintillation vials (Sarstedt, Nümbrecht, Germany) (Table 1). Each vial contained 1 g (DM) of fibers. Hydrolysis reaction was initiated by applying a certain volume of enzymatic or acid solution to the fibers, which were placed in the reaction vials. At 25% w/v substrate concentration, the substrate absorbed most of the solution and had the consistency of a wet material, that is, not a suspension. Electrical insulation tape was used to seal plastic screw caps to the vials in order to prevent caps from opening and causing leakage of hydrolysates. Unless stated otherwise, enzymatic and acid hydrolyzes were carried out in duplicates, at 50°C and at 25% substrate concentration. Enzymatic hydrolysis was terminated by submerging plastic scintillation vials into boiling water for 10 min. Acid hydrolysis, on the other hand, was stopped by neutralizing hydrochloric acid with 2 M NaOH and 50 mM Na‐Citrate buffer (pH 5) solutions.

**TABLE 1:** Overview of experimental process used to investigate attrition and saccharification of flax fibers

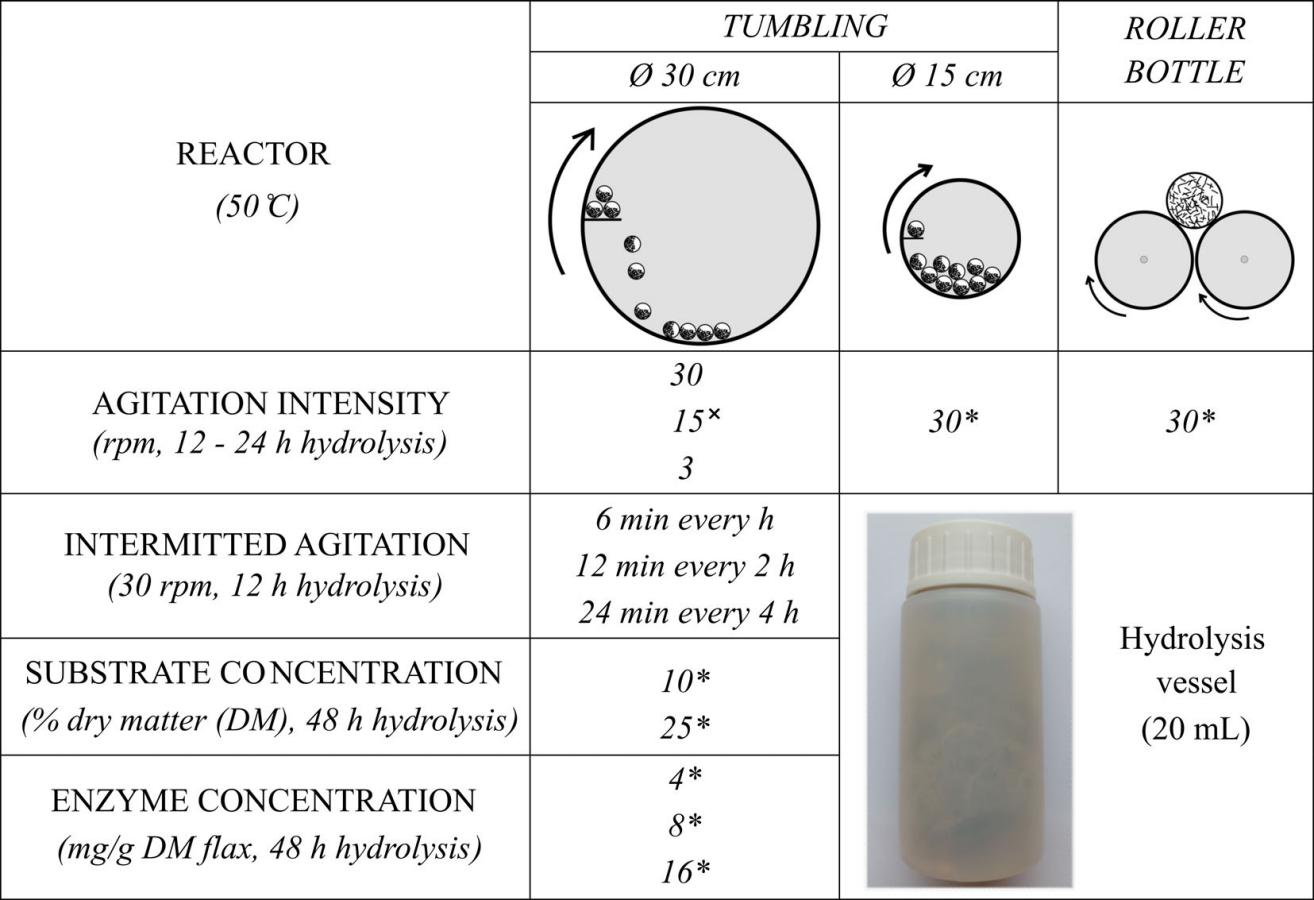

*Note:* Symbols * and ˣ indicate that hydrolysis was carried out using enzymes or HCl acid, respectively. Twenty milliliters hydrolysis vessels were subjected to tumbling inside 30 or 15 cm diameter reactors. The length of the tumbling reactors was the same—37 cm. The size (diameter) of the bottles schematically shown inside the tumbling reactors is kept proportional to the size (diameter) of the reactors and the length of integrated paddles. In the roller bottle reactor, bottles were not subjected to tumbling, but instead were horizontally oriented and rotated around their axis.

### 2.1 Effect of reactor design and operation

Three different laboratory‐scale agitation systems were used: a *roller bottle reactor* and two horizontally rotating *tumbling reactors* (Table 1). The latter two only differed in the drum diameter (30 and 15 cm) and in the length of the integrated paddle (5 and 2.5 cm, respectively) that, during rotation, lifted and dropped the bottles placed within the drums (37 cm in length).^26^ In the roller bottle reactor, bottles were not subjected to tumbling, but instead were horizontally oriented and rotated around their axis at 30 rpm. Three different rotation speeds (30, 15, and 3 rpm) of the tumbling reactor (Ø 30 cm) were applied to investigate the influence of agitation intensity on flax fiber attrition. The effect of intermittent tumbling on fiber attrition was investigated by mixing interruptedly at certain time points: (i) for 6 min each hour; (ii) for 12 min every 2 hr and (iii) for 24 min every 4 hr. The intermittent agitation (30 rpm) was applied at the end of each hydrolysis period (e.g., agitation for 6 min was initiated after 54 min of stationary hydrolysis). At the onset of hydrolysis, all reaction vessels were agitated at 30 rpm for a duration of 5 min to uniformly redistribute enzymes/acid within the substrate. Unlike for enzymatic hydrolysis, flax fibers were pre‐incubated with hydrochloric acid solution for 12 hr before initiating the intermitted mechanical treatment.

### 2.2 Effect of enzyme loading and substrate concentration

To investigate the effect of enzyme and substrate concentration on flax fiber attrition and saccharification, three doses of Cellic CTec2 enzyme preparation (4, 8, and 16 mg protein/g flax) and two doses of substrate loading (25 and 10% DM) were tested (Table 1).

### 2.3 Analytical methods

Flax fiber dimensions were analyzed with a PulpEye (Eurocon Analyser AB, Sweden) particle analyzer in manual mode.^26^ Prior to each measurement, all non‐hydrolyzed fibers (<1 g) were transferred into a beaker containing 3 L of tap water. In order to disintegrate flax fiber agglomerates, diluted fiber suspensions were then gently agitated with a rubber spatula. The settings of the instrument were such that approximately 100,000 individual particles in each sample were measured, that is, not all the fibers present in each sample. Data analysis of the imaged particles was carried out using MATLAB R2014a software (MatWorks, Natick, MA). Before the analysis, fiber data results from two replicates were pooled together.

The chemical composition of flax fibers was determined following published protocols.^27^ A Dionex ICS5000 HPAEC system equipped with pulsed amperometric detector (Dionex, Sunnyvale, CA) and a CarboPac PA1 2 × 250 mm analytical column was used to quantify sugars released during acid hydrolysis. The instrument was operated at a flow rate of 0.25 ml/min at 30°C and with the elution gradient of pure water for 32 min followed by a linear gradient from 0 to 0.25 M NaOH for 5 min, and a constant flow of 0.25 M NaOH for 4 min. Initial conditions were then restored in 3 min and the system was finally allowed to recondition with pure water for 10 min.

Soluble enzymatic hydrolysis products in the saccharification reactions were quantified with an Ultimate 3000 HPLC (Dionex, Germering, Germany) coupled with a refractive index detector (Shoden, Japan). The flow rate was 0.6 ml/min at 80°C using 5 mM H_2_SO_4_ as mobile phase. Twofold dilution of the hydrolysates with ultra‐pure water was applied prior to HPLC analysis.

## 3 RESULTS AND DISCUSSION

### 3.1 Effect of reactor design

**FIGURE 1:**
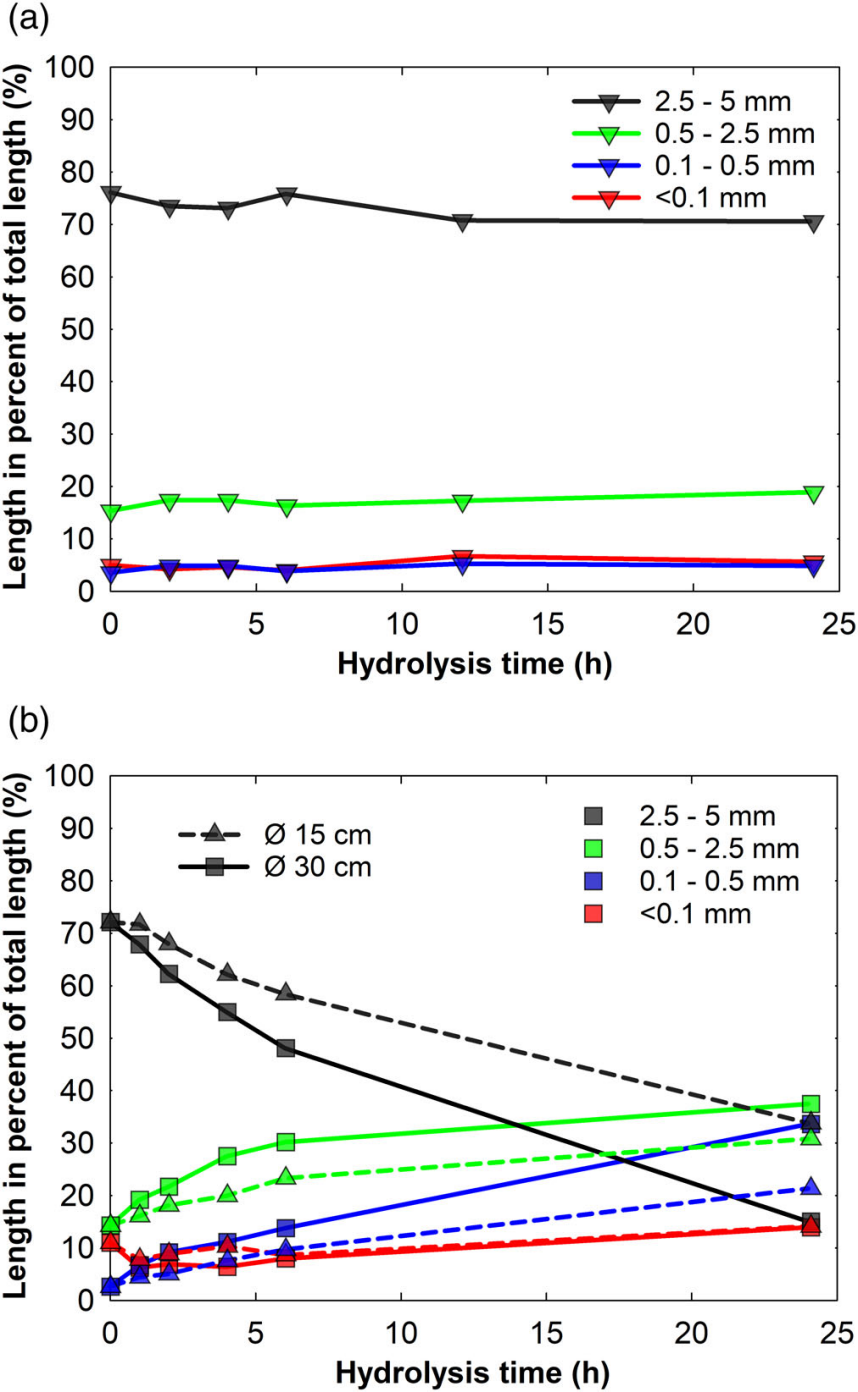
Effect of (a) roller bottle setup and (b) tumbling reactor diameter on flax fiber attrition during enzymatic hydrolysis. In (b), solid and dashed lines represent changes in fiber length distributions when hydrolysis was carried out in the tumbling reactors having drum diameters of 30 and 15 cm, respectively. Four different colors of symbols and lines are used to represent different fiber length ranges

The flax fiber attrition data obtained from three different reactor setups (Table 1) is shown in Figure 1 where fiber length distribution is divided into four classes of fiber length ranges: 2.5–5 mm, 0.5–2.5 mm, 0.1–0.5 mm, and <0.1 mm. At each time point the sum of all fiber lengths is 100%. The fiber length data presentation in percent of total length of all fibers is well suited to represent the long fiber contribution more fairly. Even though great care was taken to remove small fibers from the substrate, for example, at the onset of hydrolysis (0 hr), for instance, the average fiber length was app. 0.1 mm. The mass and volume of the substrate, however, were dominated by long fibers and fiber bundles. This could be illustrated by the fact that 400 fibers of 5 mm alone match 20,000 fibers having a length of 0.1 mm, yet it is known that the long fibers are the most important for the viscosity.^22^ As illustrated in Figure 1, at the onset of hydrolysis the substrate consisted mostly of long fibers ranging from 2.5 to 5 mm (app. 75% of total fiber length). It can be noted that in contrast to tumbling (subplot B), the rotational agitation had no major effect on fiber attrition (subplot A) (Figure 1). The substantial differences in fiber attrition patterns confirm that reactor design plays an important role in biomass degradation.

When comparing the tumbling reactor designs (Figure 1b), the twofold reduction in reactor diameter (from 30 to 15 cm) resulted in slower attrition of long, that is, 2.5–5 mm fibers and at the same time the share of intermediate (i.e., 0.5–2.5 mm) and short (i.e., <0.5 mm) fibers increased at a slower pace.

The magnitude of the impact force to which fibers are subjected when the bottles hit the bottom side of the reactor is greater for a longer fall distance, see Equation (1). A faster fiber shortening in a reactor having a larger diameter is therefore expected. The impact force dependence on fall height is illustrated in Table 2 for two different substrate loadings when the same amount of a substrate is used and assuming that all water is associated with the substrate and the slowdown distance is the same, that is, 2 cm. Noteworthy, the impact force scales up proportionally with the increasing fall height. Due to the higher hydrolysate mass, impact force is higher when hydrolysis is carried out at 10% substrate concentration. Free water which is not associated with a hygroscopic lignocellulosic substrate is, however, often present at 10% substrate loading. Free water certainly affects the slowdown distance and, as a result, the correlation of the impact force with the hydrolysate mass is not straightforward.

**TABLE 2:** Impact force dependence on fall height illustrated for two different substrate loadings (10 and 25%) when the same amount of a substrate is used (1 g) and assuming that all water is associated with the substrate and a slow down distance is the same, that is, 2 cm

Substrate concentration (%)	10	10	25	25
Total mass of hydrolysate (kg)	0.01	0.01	0.004	0.004
Gravitational acceleration (m/s^2^)	9.8	9.8	9.8	9.8
Slow down distance (m)	0.02	0.02	0.02	0.02
Reactor diameter/fall height (m)	0.15	0.3	0.15	0.3
Impact force (N)	0.735	1.47	0.294	0.588

It is important to emphasize that all hydrolysis experiments reported here were carried out in plastic vessels, which undoubtedly also absorbed part of the impact energy during tumbling. Moreover, the fall height of the vessels did not correspond to the actual reactor diameter. The paddle that lifted the bottles was designed in such a way that the bottles started to fall when they reached approximately half of the drum height. Furthermore, the number of bottles within the two different reactors was the same. Twofold decrease in fall height, thus, was not directly experienced by the substrate, as the specimen crowding within the reactor of smaller dimensions was much higher. During tumbling the substrate inside the reaction vessels was not only exposed to the impact force, but also to the rotational movement which occurred when vessels rotated at the bottom of the drum, before being picked up and lifted by the integrated paddle. It is difficult to estimate the extent of his rotational event, since the rotational movement of each individual bottle was partially restricted by neighboring bottles and also limited by a limited friction between the metal surface of the drum and the bottles. This meant that the bottles inside the drum tended to slide down the drum wall without rotating when the drum rotated. The effect of the rotational agitation on fiber attrition was likely different from the roller bottle experiment, since tumbling induces agglomeration of flax fibers.^9^ This leads to a substantial reduction of the substrate volume within the reaction vessels, and in such a way permits easier substrate movement within the vessel. Due to the aforementioned effects it is challenging to precisely estimate the magnitude of impact force to which fibers were exposed during tumbling.

The roller bottle results from this study are in contrast with those of Roche et al who found that rotational mixing within 125 and 250 ml reaction vessels lead to efficient liquefaction of dilute sulfuric acid pretreated corn stover, even at 30% substrate loading.^16^ The present observation of limited fiber attrition (Figure 1a) as well as absence of fiber agglomeration (visual observation) indicate that forces acting during rotational mixing were insufficient to induce fiber breakage or even a movement of relatively long flax fibers within the 20 ml reaction vessels, that is, they were insufficient to overcome the strength of the fiber network and its association with the vessel surface. Noteworthy, the large void volume required to achieve efficient biomass mixing within tumbling type reactors is a considerable drawback of this reactor system.^2^ The contrasting outcome of the two studies can potentially be attributed to the different void volumes within the reaction vessels, but also to the specific biomass properties, for example, fiber morphology, initial fiber length distribution, chemical composition, as well as the reaction conditions, that is, substrate concentration, reactor and enzyme specifics.^16^

In this study, it was also attempted to vary the mass of the hydrolysate within the reaction vessels, that is, 5 or 10 g, in order to increase the impact force during the tumbling agitation in the reactor equipped with the 30 cm drum diameter. Unlike reactor dimensions (free fall distance) hydrolysate mass had no major effect on fiber attrition patterns (data not shown). Noteworthy, increase in the hydrolysate mass directly resulted in an increase of the amount (i.e., from 1.25 to 2.5 g, at 25% substrate concentration or from 0.5 to 1 g, at 10%) as well as in volume of the substrate within the reaction vessels. Substantial substrate volume changes within the reaction vessels most likely had a pronounced effect on the strength of the flax fiber network within the reaction vessel as well as fiber network interaction with the vessel surface. Both these factors possibly affected slow down distance and reduced the forces transmitted to the fibers during impact, resulting in a “softer” fiber deformation despite the higher mass (Equation (1)).

In summary, the results suggest that fall height is the most decisive parameter of the reactor design with regards to fiber attrition.

### 3.2 Effect of agitation intensity

The effect of mixing intensity on the attrition of flax fibers was examined by reducing agitation rate 10 times, from 30 to 3 rpm. During the acid hydrolysis, mixing was also carried out at 15 rpm.

**FIGURE 2:**
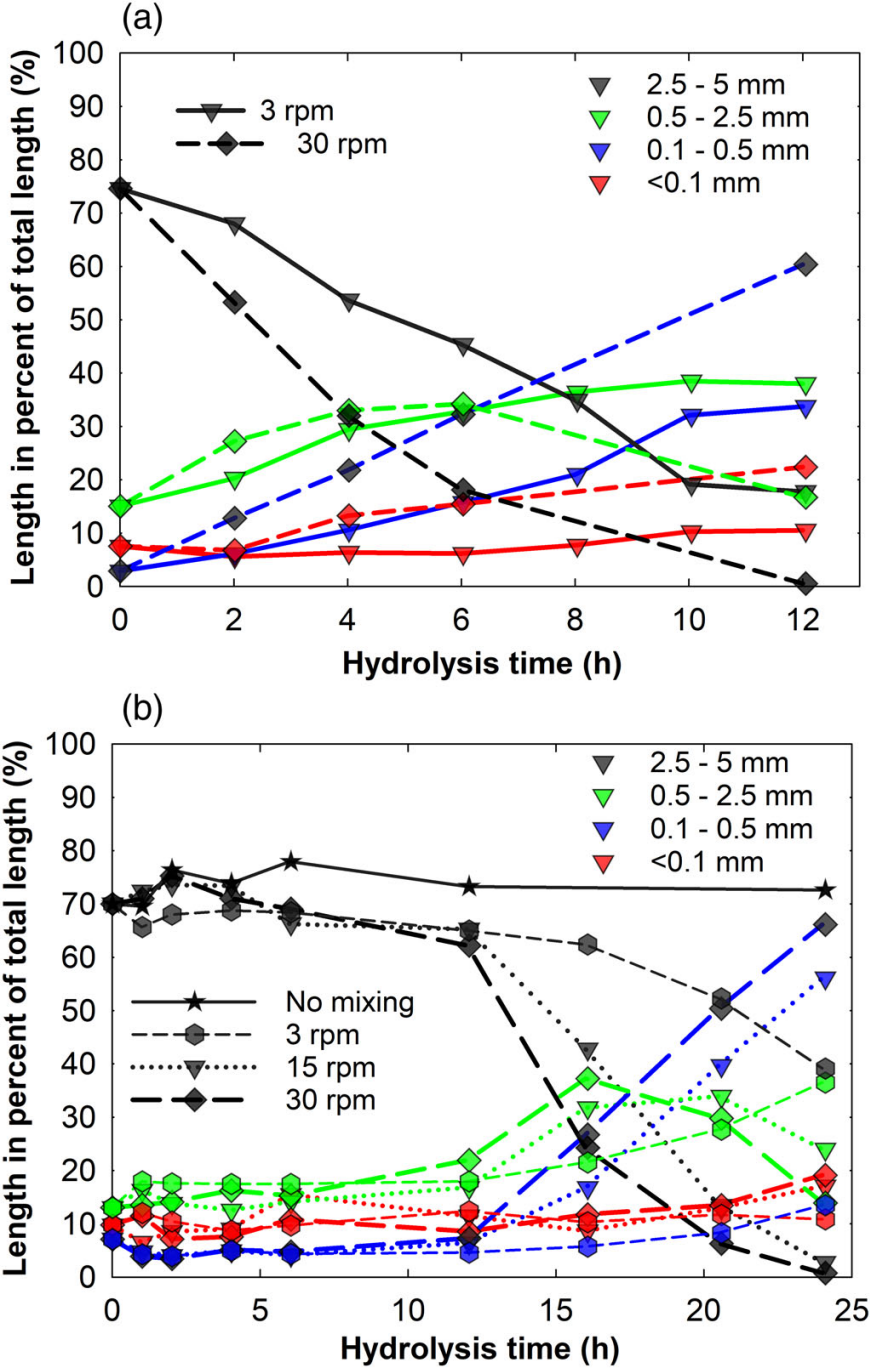
Effect of mixing intensity on flax fiber attrition during (a) enzymatic and (b) acid hydrolysis. Hydrolysis experiments were carried out at 25% substrate loading in the tumbling reactor having a diameter of 30 cm. The enzyme loading during hydrolysis was 16 mg/g DM flax. In subplot (b), single black line with stars represent attrition of the fibers ranging between 2.5 and 5 mm when acid hydrolysis was carried out without agitation. Four different colors of symbols and lines are used to represent different fiber length ranges

From Figure 2a, it can be noted that tenfold reduction in agitation intensity during enzymatic hydrolysis considerably slowed down attrition of flax fibers. Agitation intensity during the acid hydrolysis impacted fiber breakage in a similar manner where both twofold (from 30 to 15 rpm) and tenfold (from 30 to 3 rpm) reductions in agitation intensity resulted in progressively slower attrition rates of flax fibers (Figure 2b). During the time period studied, acid hydrolysis without agitation did not result in substantial fiber attrition (only a share of long, i.e., 2.5–5 mm fibers is shown in Figure 2b). This finding is in agreement with previously published data showing that enzymatic hydrolysis without agitation, although it leads to significant hydrolysis yields, do not induce considerable attrition of flax fibers.^9^, ^28^

It is worth noting that extensive fiber attrition during acid hydrolysis only occurred after a delay phase of app. 12 hr, unlike for simultaneous enzymatic and mechanical treatment. The long stagnant phase seen before the fast fiber attrition is likely a result of physical and catalytic differences between the catalysts. H_3_O^+^ ions are significantly smaller, ~0.100 nm,^29^ than enzymes (typical cellulase has a diameter of 4–6.5 nm and is 18–21.5 nm long),^30^ and can therefore diffuse and penetrate plant cell walls more evenly than enzymes. We speculate that this implies that it takes longer time for a sufficient degree of hydrolysis to take place in locations prone to breakage. Enzymes are on the other hand limited to diffusing into larger pores or other defects in the cell walls.^31^

The results above indicate that there is a simple relationship between agitation speed and the rate at which attrition happens during enzymatic hydrolysis. The design challenge is thus to find an agitation speed that gives sufficiently fast liquefaction without inducing unacceptable deactivation rates of the enzyme preparation used.

### 3.3 Effect of intermittent tumbling

Intermittent tumbling was tested as an attempt to introduce a milder, but still efficient, agitation scheme. Intermittent tumbling during enzymatic or acid hydrolysis was carried out following three different schedules: (i) for 6 min each hr, (ii) for 12 min every 2 hr, or (iii) for 24 min every 4 hr in the tumbling reactor, Ø 30 cm, rotating at a speed of 30 rpm. This implies that independently of the agitation intervals used, samples were exposed to the same magnitude of mechanical treatment over a 12 hr period (i.e., 2,160 reactor revolutions in total), which also corresponds to the agitation intensity obtained during continuous mixing at 3 rpm. In these experiments, higher enzyme loading, 16 mg/g of substrate, was used.

**FIGURE 3:**
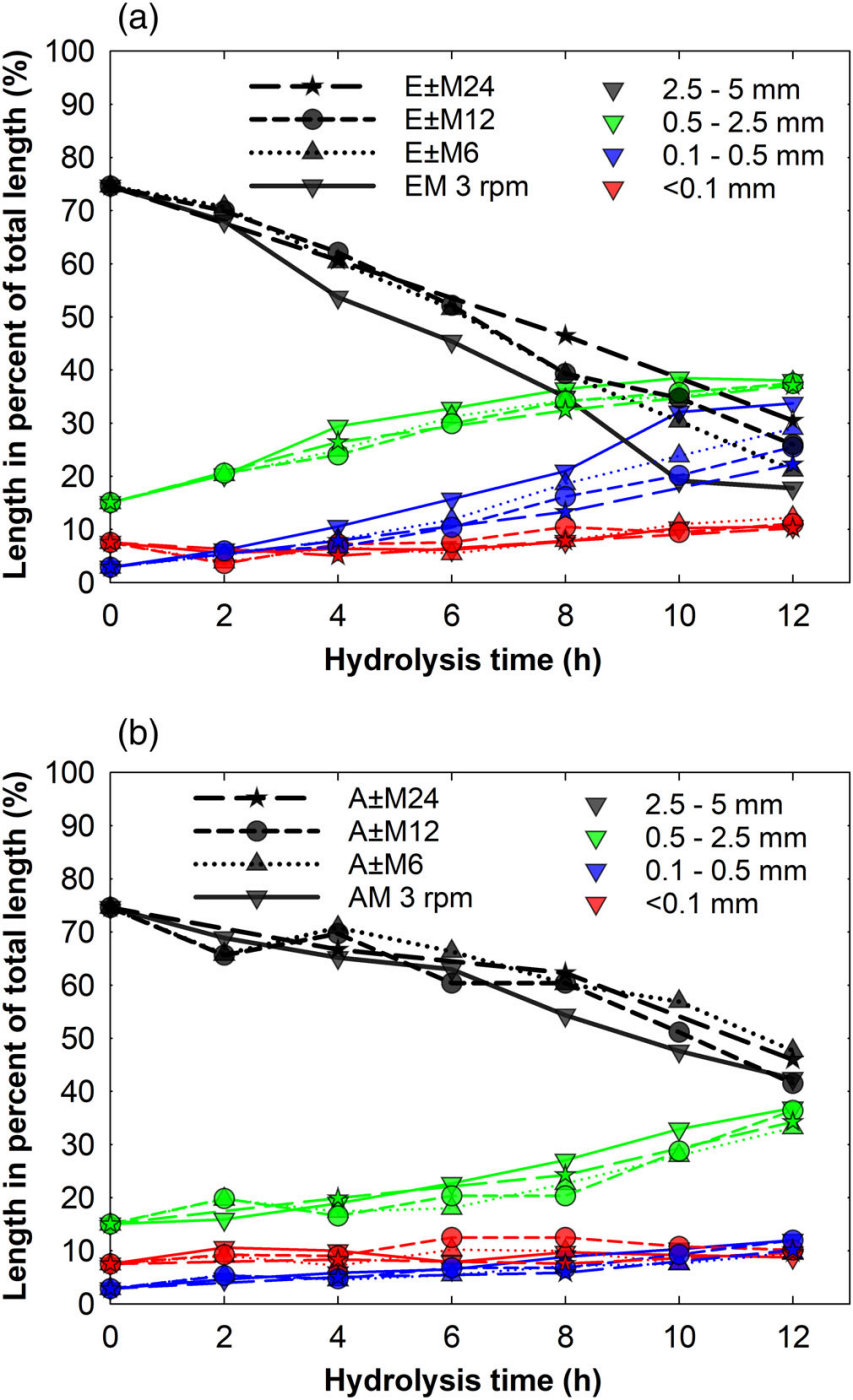
Effect of intermittent tumbling on flax fiber attrition during (a) enzymatic and (b) acid hydrolysis. EM, AM (solid lines) and E ± M, A ± M (dashed and dotted lines) indicate that hydrolysis (either enzymatic (E) or acid (A) was carried out simultaneously or mixing (M) was applied interruptedly at certain time periods, respectively. Magnitude of the mechanical agitation over the 12 hr period was the same in all cases—2,160 reactor revolutions. The enzyme loading during hydrolysis was 16 mg/g DM flax. Flax fibers were preincubated with 1.6 M HCl acid solution for 12 hr before initiating intermittent treatment. Four different colors of symbols and lines are used to represent different fiber length ranges

It was found that even though the total number of reactor revolutions was the same in all cases, the fiber attrition profiles during enzymatic hydrolysis were different (see Figure 3a). The attrition of flax fibers turned out to be progressively slower with increasing time periods between agitation cycles. The trend is best represented by attrition of long, that is, 2.5–5 mm flax fibers. Noteworthy, the continuous mixing at 3 rpm accelerated flax fiber attrition the most. The observed difference in fiber attrition during enzymatic hydrolysis cannot be attributed to the mechanical forces since the magnitude of agitation, that is, the fatigue damage due to cyclic loading during the intermittent tumbling in all instances was the same. Contrary to the enzymatic‐intermittent tumbling, the trends of fiber attrition profiles were somewhat similar during acid‐intermittent tumbling (Figure 3b). Therefore, other factors, most likely related to the enzymes, lead to the aforementioned differences. During the intermitted tumbling, enzymes are given periods of time to hydrolyze the substrate undisturbed by mechanical forces.^9^ Processive enzymes, such as typical cellobiohydrolases, remain active unless they are physically obstructed, for example by lignin, and continue to hydrolyze cellulose even in the absence of mixing.^32^ A static hydrolysis has its benefits since it has been shown that mechanical agitation may reduce enzyme activity.^9^ Lack of agitation, on the other hand, also leads to accumulation of reaction products in the vicinity of enzymes (end product inhibition) and may lead to a lack of redistribution of non‐processive enzymes, the diffusion of which toward accessible substrate areas potentially becomes limited. Endoglucanases, for instance, are often non‐processive enzymes, but they play a key role in fiber attrition.^22^ Endoglucanases are known to preferentially attack less crystalline cellulose regions (often called amorphous regions) and break β‐(1,4)‐glycosidic linkages internally.^33^ The regions in plant fibers where cellulose microfibrils are less ordered, that is, dislocations, are often crack initiation sites during mechanical loading.^34^ Consequently, endoglucanases additionally weaken these fiber regions and makes fibers more vulnerable to mechanical failure.^17^, ^35^ In a recent study, Ciesielski et al employed direct nano‐manipulation of cellulose nanofibrils and showed that fibrils can be kinked by applied mechanical force to such an extent that breakages in the glucan chains are introduced, which then provides initiation sites for processive, exo‐active cellobiohydrolases (i.e., Cel7A).^36^ The study further supports the finding that enzymes and mechanics act synergistically in fiber attrition. The progressively slower fiber attrition seen during the intermittent tumbling suggests that such agitation is less efficient than continuous, but slower, agitation in ensuring redistribution of enzymes, in particular, endoglucanases.

As described previously, prior to intermittent tumbling the fibers where subjected to 12 hr static acid hydrolysis. This step was introduced because no significant fiber attrition occurred during the initial 12 hr of simultaneous acid and mechanical treatment (Figure 2b).

Data in Figure 3 suggest that in order to achieve fast fiber attrition, continuous rather than intermitted agitation should be applied during enzymatic hydrolysis of lignocellulose.

### 3.4 Effect of enzyme concentration

**FIGURE 4:**
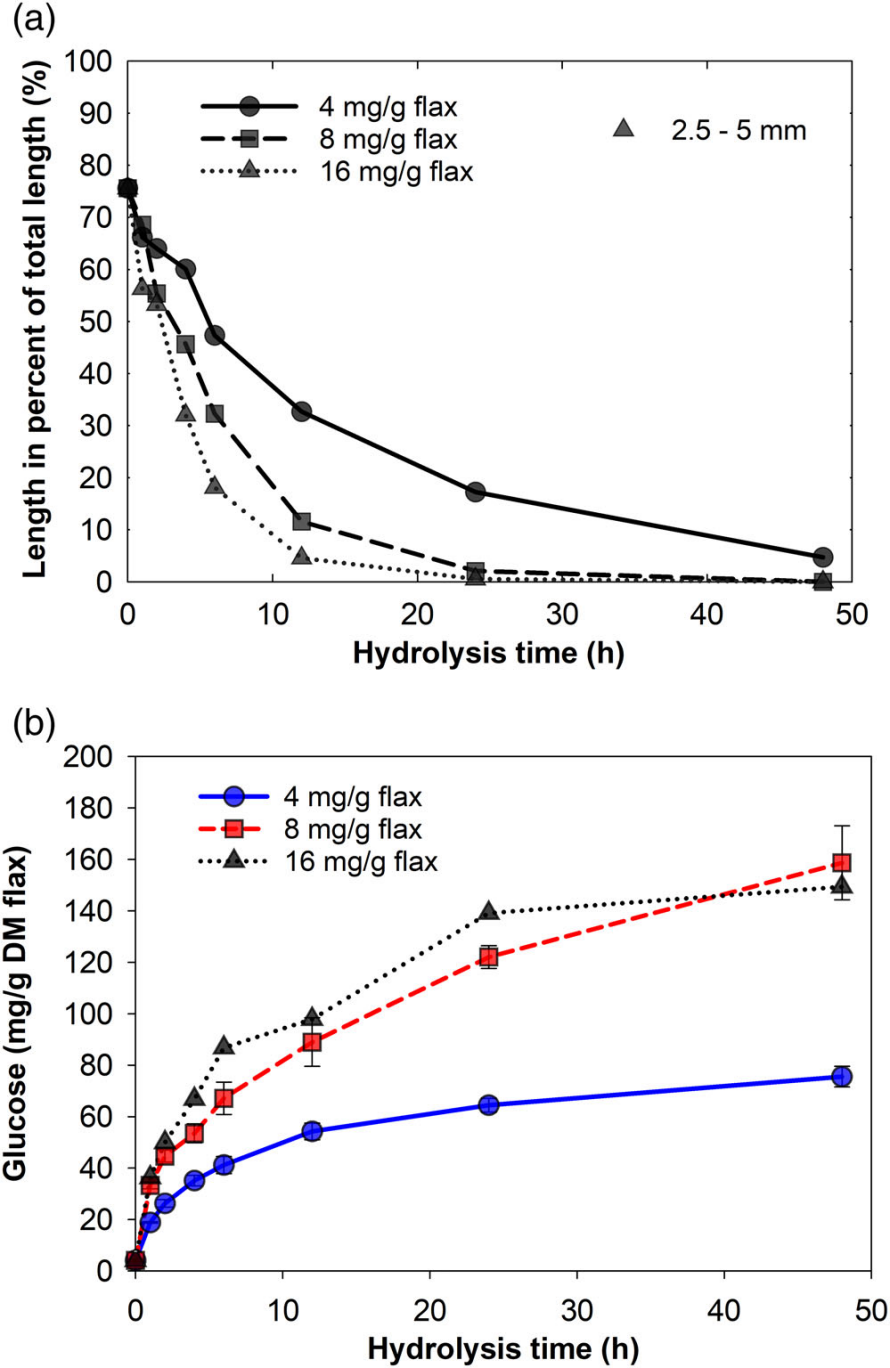
Effect of enzyme concentration on (a) flax fiber attrition and (b) saccharification rate. In (a), only lines showing the development of the fibers ranging between 2.5 and 5 mm are plotted. Please note that error bars in the subplot (b) represent SD of only two measurements. Glucose release during hydrolysis with the highest enzyme loading (16 mg/g DM flax) is represented by a single replicate

Data in Figure 4a,b indicate that fiber attrition as well as saccharification could be considerably promoted by increasing enzyme loading. When the enzyme concentration was increased from 4 mg/g DM to 8 mg/g DM flax, both improved fiber attrition and higher hydrolysis yield were obtained. A further increase in enzyme concentration to 16 mg/g DM substrate, on the other hand, had a very limited effect on these processes. Hydrolysis yield can be directly associated with the depolymerization of structural polymers (i.e., holocellulose) and thus a physically weaker fiber structure. Therefore, the result that increasing enzyme loading boosts fiber attrition is predictable. In agreement with our results, Szijártó et al observed that higher enzyme concentration during hydrolysis of hydrothermally pretreated wheat straw results in a faster reduction of hydrolysate viscosity.^37^

The phenomenon that increasing enzyme concentration enhances holocellulose conversion only to a certain extent has been noted previously.^13^, ^23^, ^38^ The absence of accessible surface areas of the substrate, that is, lack of potential enzyme binding sites, has been proposed to be responsible for the observed retardation of hydrolysis rate at high enzyme loading.^23^ Dissociation of flax fiber bundles and fragmentation of fibers, as seen in Figure 4a, during the treatment potentially increased accessible surface area of the substrate. Yet, the result that similar glucose yields were obtained when the enzyme concentration was either 8 mg/g DM or 16 mg/g DM flax, indicates that enzymes at these hydrolysis conditions were in excess.

### 3.5 Effect of substrate concentration

**FIGURE 5:**
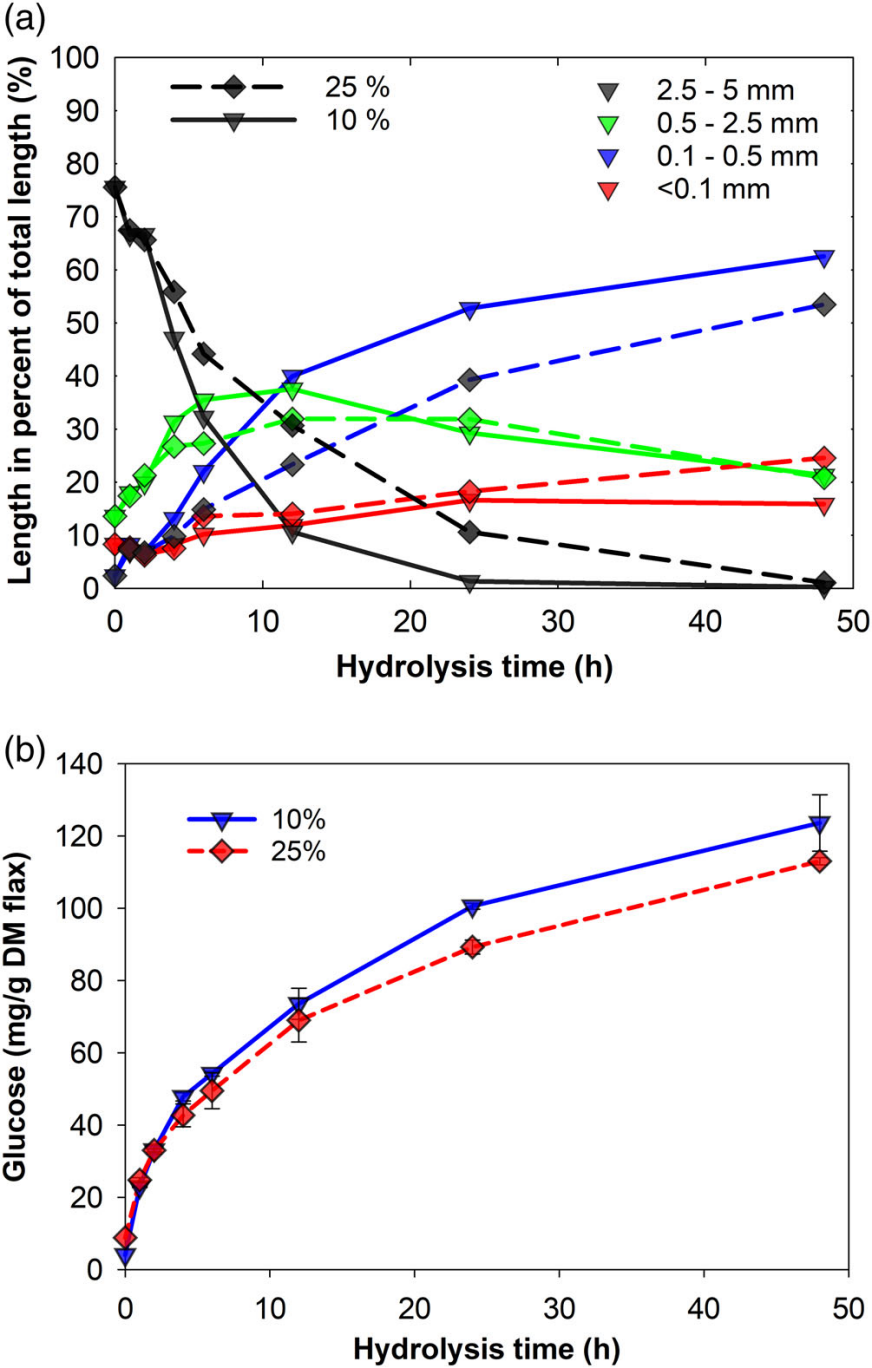
Effect of substrate concentration on (a) flax fiber attrition and (b) saccharification rate during enzymatic hydrolysis at 10% and 25% (w/w) solid loadings. Please note that error bars in the subplot (b) represent SD of only two measurements. Four different colors of symbols and lines are used to represent different fiber length ranges

The substrate concentration was another factor that considerably influenced fragmentation and saccharification of flax fibers, Figure 5a,b, respectively. A decrease in the substrate concentration from 25 to 10% w/v resulted in considerably faster fiber attrition as well as higher hydrolysis yield, that is, glucose concentration was 12% higher after 24 hr of hydrolysis. The effect that increasing lignocellulose concentration during hydrolysis negatively affects hydrolysis yield has been routinely observed in similar studies.^2^, ^7^, ^39^ It has been suggested that reduced enzyme mobility through the medium containing limited amounts of free water, changing rheological properties, heat and hydrolysis product transfer, end‐product inhibition, as well as other factors could be responsible for the observed decrease in saccharification rates.^7^, ^13^, ^40^ However, more recent studies have pointed out that the relationship between sugar yields and solids contents may be less straight forward than hitherto assumed and for example depends on the enzyme preparation used.^4^

## 4 CONCLUSIONS

It was found that both reactor design, for example, the diameter of tumbling type reactors, as well as operating conditions, that is, rotational speed and intermittent mixing, affect the attrition rate of flax fibers during hydrolysis. Different fiber attrition patterns were observed despite the same mixing intensities applied during enzymatic and acid hydrolysis experiments, indicating that enzymes target and damage lignocellulose more selectively and less evenly than hydrochloric acid. Results confirmed that increased enzyme concentration (to a certain limit) as well as reduced substrate loading during hydrolysis leads to faster fiber attrition and higher saccharification yields. To summarize, the findings of this work indicate that reactor design and operation as well as hydrolysis conditions are all important factors, which needs to be optimized meticulously to obtain efficient lignocellulose depolymerization and low operating costs of a biorefinery facility. The results suggest that fiber attrition, and thus liquefaction, in biorefineries can be improved by increasing the fall distance of a fibrous substrate within a reactor, which means the need to increase the void volume. This, within existing tumbling reactors, for instance, may be achieved by reducing the amount of substrate within the reactor.

## CONFLICT OF INTEREST

The authors declare no conflict of interest.

## AUTHOR CONTRIBUTIONS

All authors participated in the conception of the original study. Ramūnas Digaitis performed all the experiments and analyzed data. Emil Engelund Thybring and Lisbeth Garbrecht Thygesen advised on experimentation throughout the progress of the study. Ramūnas Digaitis wrote original draft of the article. All authors critically revised the draft and approved the final manuscript.

5

### PEER REVIEW

The peer review history for this article is available at https://publons.com/publon/10.1002/btpr.3083.
